# Integration of MRI and somatosensory evoked potentials facilitate diagnosis of spinal cord compression

**DOI:** 10.1038/s41598-023-34832-2

**Published:** 2023-05-15

**Authors:** Shu-Pin Sun, Chun-Ren Phang, Shey-Cherng Tzou, Chang-Mu Chen, Li-Wei Ko

**Affiliations:** 1 International Ph.D. Program in Interdisciplinary Neuroscience (UST), College of Biological Science and Technology, National Yang Ming Chiao Tung University, 734, Engineering Bldg. 5, 1001 Daxue Road, Hsinchu, 30010 Taiwan, ROC; 2 Department of Medical Research, National Taiwan University Hospital Hsin-Chu Branch, Hsinchu, 300 Taiwan, ROC; 3 Institute of Molecular Medicine and Bioengineering, National Yang Ming Chiao Tung University, Hsinchu, 300 Taiwan, ROC; 4 Department of Surgery, College of Medicine and Hospital, National Taiwan University, No. 7, Zhongshan South Road, Taipei, 10002 Taiwan, ROC; 5 Center for Intelligent Drug Systems and Smart Bio-devices (IDS2B), College of Biological Science and Technology, National Yang Ming Chiao Tung University, Hsinchu, 300 Taiwan, ROC; 6 Institute of Electrical and Control Engineering, Department of Electronics and Electrical Engineering, National Yang Ming Chiao Tung University, Hsinchu, 300 Taiwan, ROC; 7 Department of Biomedical Science and Environment Biology, and the Drug Development and Value Creation Research Center, Kaohsiung Medical University, Kaohsiung, 807 Taiwan, ROC

**Keywords:** Neuroscience, Diseases, Medical research, Neurology

## Abstract

This study aimed to integrate magnetic resonance imaging (MRI) and related somatosensory evoked potential (SSEP) features to assist in the diagnosis of spinal cord compression (SCC). MRI scans were graded from 0 to 3 according to the changes in the subarachnoid space and scan signals to confirm differences in SCC levels. The amplitude, latency, and time–frequency analysis (TFA) power of preoperative SSEP features were extracted and the changes were used as standard judgments to detect neurological function changes. Then the patient distribution was quantified according to the SSEP feature changes under the same and different MRI compression grades. Significant differences were found in the amplitude and TFA power between MRI grades. We estimated three degrees of amplitude anomalies and power loss under each MRI grade and found the presence or absence of power loss occurs after abnormal changes in amplitude only. For SCC, few integrated approach combines the advantages of both MRI and evoked potentials. However, integrating the amplitude and TFA power changes of SSEP features with MRI grading can help in the diagnosis and speculate progression of SCC.

## Introduction

Studies have shown that patients with spinal cord compression (SCC) experience neurological impairment and deterioration caused by the gradual narrowing of the spinal canal^1–5^. However, not all patients with SCC experience symptoms, even SCC disease progression can produce different symptoms from patient to patient, and no evidence supports the management of its indicators^6–8^; thus, these issues suggest the limited understanding of SCC progression. Therefore, given the increased prevalence of SCC and associated increased disability and cost, improved diagnostic evaluation of patients suspected of SCC is needed for early detection and intervention before the occurrence of irreversible histological damage to the spinal cord and for the prevention of severe permanent disability^7,9–11^. Therefore, in the course of SCC-induced myelopathy, the degree of neurological impairment and progression should be determined and monitored, so that clinicians can adequately counsel patients and initiate appropriate treatment modalities^9,12^.

In clinical practice, SCC is often treated with surgical decompression based on magnetic resonance imaging (MRI) findings and symptoms^13,14^. MRI can provide physical structural information about the location, size, etc., of SCC^5^; however, current studies have shown that relying solely on traditional MRI scans is insufficient to accurately predict the severity of neurological impairment^15–18^. With the development of advanced MRI techniques such as diffusion MRI and multiparametric quantitative MRI, they have been studied as potential tools for monitoring the progression of SCC and evaluating treatment outcomes^19–21^. Neurological examination and functional assessment scales are commonly used to assess the clinical symptoms of patients with myelopathy, but imaging reports have found mild-to-moderate stenosis in asymptomatic cases^22^. In older patients, clinical complaints, presentations, etc., are often confused with symptoms of peripheral neuropathy or musculoskeletal pain. Because neurological examination mainly relies on the patient’s perception of symptoms and subjective findings of the examiner, they lack sensitivity and may not reflect subtle changes in the degree of neurological damage^10,23–25^ which affects counseling and treatment planning. Studies have suggested that MRI and neurophysiological evoked potentials (Eps) may hold promise for the quantification of spinal cord tissue damage for early detection, monitoring disease progression, and prognosis prediction^3,9,26,27^. Therefore, the combination of MRI and electrophysiological Eps may aid in the diagnosis of SCC.

Neurophysiological Eps are helpful in assessing neuropathy and are sensitive and objective ^25,26,28,29^. Among the neurophysiological Eps, somatosensory evoked potentials (SSEPs) are elicited by stimulating sensory fibers within nerves that specialize in transmitting sensory information from various parts of the body. These nerves conduct signals at a very high speed and can transmit signals between the nerve and the brain, making SSEPs useful in assessing nerve conduction velocity, sensory pathway function, spinal cord disorders, and nerve injuries. SSEPs can be evoked by stimulating the median nerve of the upper extremity or the posterior tibial nerve of the lower extremity, and then the sensory cortical responses are recorded at N20 or P40 peaks on the scalp. SSEPs can detect the function of the entire conduction pathway of the ascending sensory nerve from the peripheral nerve to the central nerve and is most commonly used for intraoperative monitoring^30–34^. A certain level of compression may damage the spinal cord, affecting SSEP features, including reduced amplitude, latency delay, or time–frequency analysis (TFA) power loss^35–39^. The voltage difference between the maximum positive and maximum negative values of the SSEP event-related potentials (ERP) response wave is the amplitude (μV), which can reflect the ability of the axon to transmit current. The conduction latency (ms) is the time measured in SSEP ERP response waveform from the start to the maximum positive voltage, which can reflect changes in the composition of nerve myelin. The TFA (mv/Hz) of SSEP can provide time, frequency, and power information. In the case of a compressed spinal cord, the TFA power is lost and has a wider distribution and high sensitivity to underlying SCC, providing an additional method of monitoring. Therefore, SSEP features may be used to identify the neurological function of patients with SCC and provide information that cannot be explained by MRI. However, for SCC diagnosis, very few studies have integrated the advantages of MRI and SSEP feature changes.

**Figure 1:**
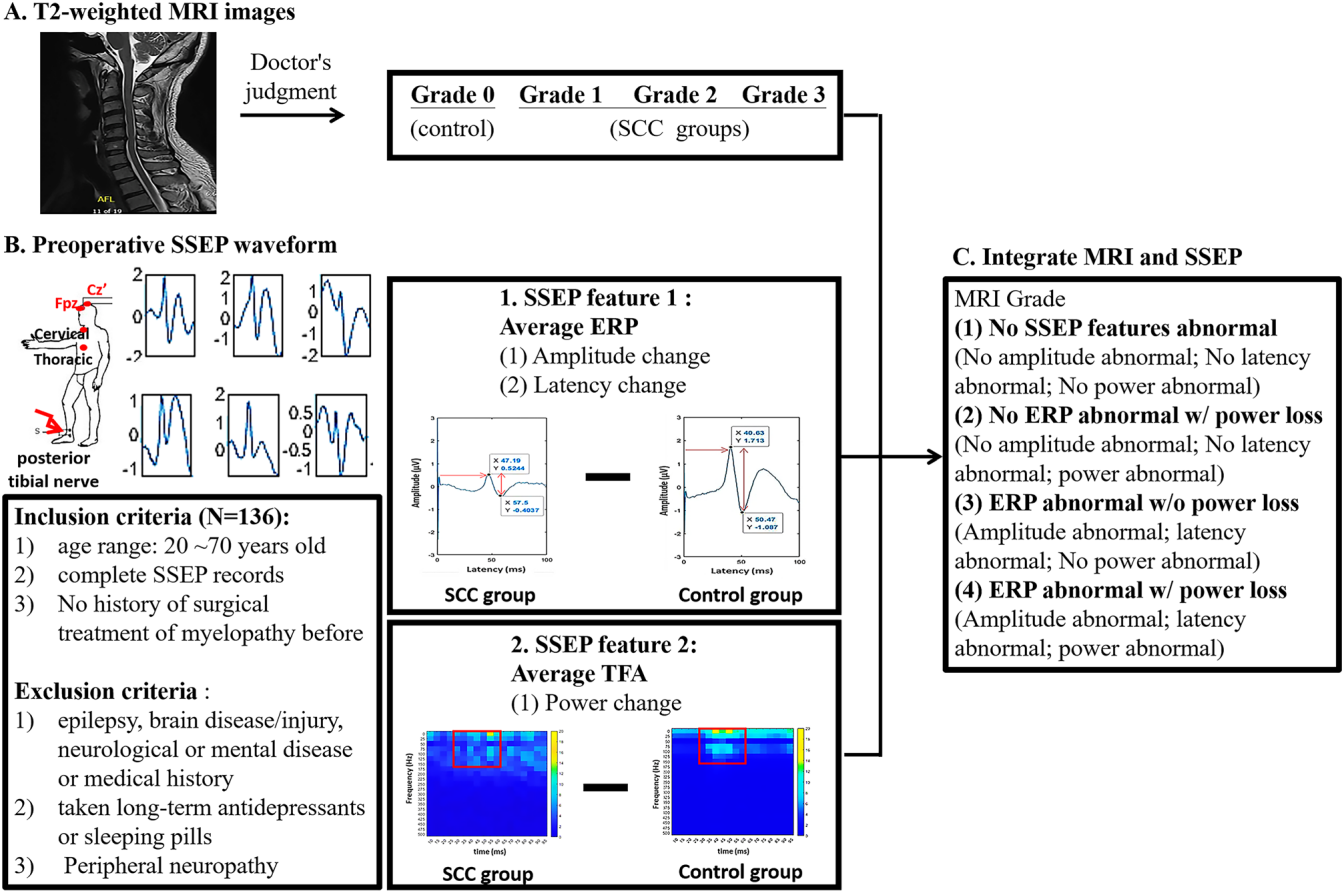
Schematic diagram of the experimental design for integrating MRI and SSEP. This retrospective study aimed to integrate MRI grades with relevant SSEP features to assist in the diagnosis of SCC. First, MRI 0–3 grading was performed according to the level of spinal stenosis (**A**), and the changes in ERPs and TFA features of preoperative SSEP were identified (**B**). Then, analyze possible SSEP feature performance under the same MRI grade for the benefit of inferred distribution of patients under various levels of spinal cord compression (**C**). ERP, event-related potential; MRI, magnetic resonance imaging; SCC, spinal cord compression; SSEP, somatosensory evoked potential; TFA, time–frequency analysis.

This retrospective study aimed to integrate the advantages of MRI and SSEP and ultimately hoped to provide more objective research evidence to reveal the clinical correlation between MRI and SSEP features to assist in SCC diagnosis. First, MRI grading was performed according to the level of spinal stenosis in patients with SCC, and the ERP and TFA features of preoperative SSEP were analyzed. An attempt was made to elucidate SCC progression by integrating the different changes in SSEP features under the same and different MRI grades (Fig. 1).

## Results

### An MRI grading system can present different levels of physical compression of the spinal cord, and SSEP feature changes varied

**Figure 2:**
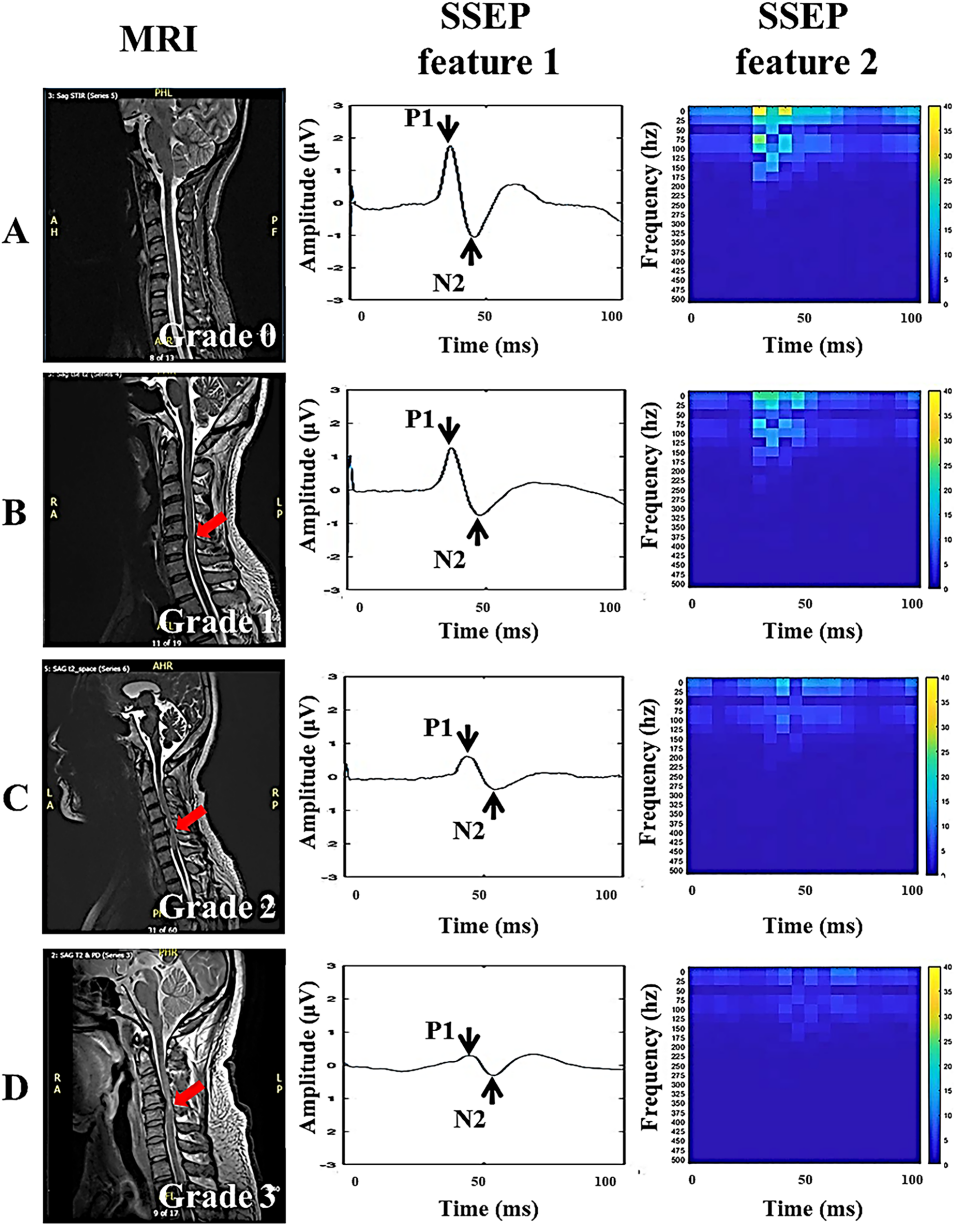
Grading of spinal stenosis using sagittal T2-weighted MRI scans. In the magnetic resonance imaging (MRI) grading of the control and SCC groups, grade 0 indicated that the normal cerebrospinal fluid (CSF) space was visible around the spinal cord, and there was no evidence of spinal cord deformity or intraspinal signal changes. This was used as the control group, which included 10 patients (**A**). Grade 1 indicated that the CSF space is occluded > 50% in the subarachnoid space, and there is no obvious spinal cord deformation. This group included 35 patients (**B**). Grade 2 referred to the compression and deformation of the spinal cord, but the MRI scan signal remains unchanged. This group included 57 patients (**C**). Grade 3 referred to marked narrowing of the spinal cord and changes in MRI scan signals. This group included 34 patients (**D**). In addition, the study shows examples of lower extremity SSEP features under each MRI grade. Red arrows show the location of spinal stenosis.

**Figure 3:**
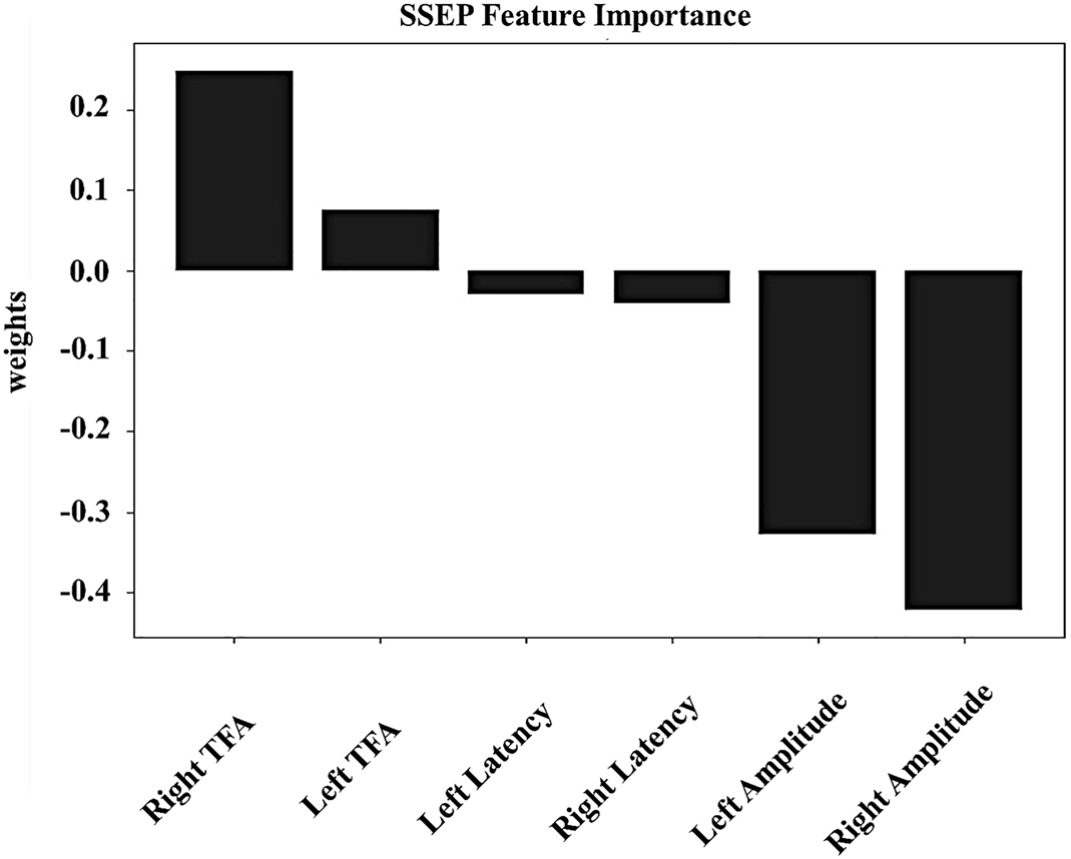
Prediction of SSEP features importance analysis. The algorithm model of QOCA aim software was used to calculate the importance of SSEP features to MRI grade. The weight analysis results showed that the importance of the right-side TFA and amplitude features was higher than that of other SSEP features.

Figure 2 shows the results of MRI grading according to the level of spinal stenosis in the control and SCC groups and examples of preoperative SSEP features extracted at each MRI grade. A total of ten patients had grade 0 (control group), thirty-five in grade 1, fifty-seven in grade 2, and thirty-four in grade 3. Table 1 shows no significant difference between MRI grading and sex, age, height, and weight, but significantly different in the results of spinal cord compression ratio (CCR) and maximum spinal cord compression (MSCC) (*p*-value < 0.05), indicating that the MRI grading method can identify different levels of physical compression and deformation of the spinal cord. In addition, significant differences were found between MRI grades and amplitude and TFA power of SSEP features. When the MRI grade was higher, the amplitude reduction and TFA power loss of SSEP features were more significant on the right side than on the left side (*p*-value < 0.01), but no difference was found in the latency of the left and right sides. In the analysis of the importance of SSEP features to MRI grading, the accuracy of the LogisticRegression operation model was 57.1%, and the weight analysis showed that the right-side TFA power (weight of 0.25) and right-side amplitude (weight of 0.33) were more important than other SSEP features (Fig. 3), and the results are similar to those in Table 1.

**Table 1:** Demographic, neuroimaging, and SSEP feature details associated with the MRI grading system.

All subjects (N = 136)	Controls (n = 10)		Compression group (n = 126)		*p*-value
Measures	Grade 0 (n = 10)	Grade 1 (n = 35)	Grade 2 (n = 57)	Grade 3 (n = 34)	
Patient demographics					
Sex (M:F)	6:4	20:15	36:21	23:11	0.839
Age (y)	52 (44, 59)	55 (48, 62)	55 (48, 61)	53 (45, 64)	0.742
Height (cm)	166.55 (157.6, 174.8)	166.40 (159.3, 170.7)	164.10 (160.0, 170.0)	165.05 (160.0, 170.4)	0.847
Weight (kg)	66.50 (54.0, 83.8)	66.00 (54.5, 72.6)	69.10 (58.0, 79.3)	71.35 (59.0, 84.0)	0.493
Image quantitative measurements					
CCR	0.48 (0.45, 0.53)	0.42 (0.40, 0.47)	0.37 (0.33, 0.42)	0.39 (0.27, 0.55)	0.000**
MSCC	2.14 (0.00, 2.86)	13.23 (8.71, 21.02)	38.38 (29.68, 53.01)	52.82 (43.52, 63.85)	0.000**
SSEP features					
Left Amplitude (mV)	2.39 (1.75, 2.80)	1.94 (1.25, 2.84)	1.40 (0.91, 2.41)	1.42 (0.81, 2.39)	0.015*
Right Amplitude (mV)	2.39 (1.67, 3.87)	1.90 (1.52, 3.13)	1.15 (0.79, 2.21)	1.00 (0.54, 2.23)	0.001**
Left Latency (ms)	40.47 (38.59, 44.38)	41.25 (39.22, 44.06)	41.56 (38.91, 45.00)	43.99 (40.16, 45.94)	0.316
Right latency (ms)	40.63 (39.22, 41.72)	41.41 (38.28, 44.84)	42.19 (39.69, 44.69)	43.76 (39.53, 48.75)	0.238
Left TFA power (mv/Hz)	12.07 (10.52, 14.73)	8.94 (7.39, 12.55)	7.56 (5.26, 12.07)	7.79 (5.62, 10.79)	0.042*
Right TFA power (mv/Hz)	12.78 (11.12, 16.53)	8.55 (6.59, 13.15)	6.60 (5.42, 11.82)	7.46 (5.20, 10.57)	0.004**

Measurements are expressed as medians (Q1, Q3). Significant differences are expressed as *p*-values, *p-value < 0.05; **p-value < 0.01. CCR, cord compression ratio; F, female; Left, left side; M, male; MRI, magnetic resonance imaging; MSCC, maximum spinal cord compression; Right: right side; SSEP, somatosensory evoked potential; TFA, time–frequency analysis.

### The correlation between amplitude and TFA power is higher than the correlation between latency

Clinically, CCR and MSCC are often used to quantitatively measure the SCC level. This study found that CCR positively correlated with left- and right-side amplitude and TFA power of SSEP features, and the difference was significant (*p*-value < 0.05), but the correlation was low (τ < 0.3), and no significant difference was found between CCR and left- and right-side latency. MSCC negatively correlated with the left- and right-side amplitude and TFA power of SSEP features and positively correlated with the right-side latency, and the difference was significant (*p*-value < 0.05); however, the correlation was low (τ < 0.3). No significant difference was found between MSCC and left latency. CCR and MSCC were associated with SSEP features, but the correlation was low, similar to previous findings^7,15–17^. In the correlation study between SSEP features, the left- and right-side amplitude and TFA power features were all correlated positively, the difference was significant (*p*-value < 0.01), and the correlation coefficient proves a moderate correlation (0.3 < τ < 0.7) between amplitude and TFA power. Latency negatively correlated with amplitude and TFA power, and the *p*-values were all < 0.01, indicating significant differences, but the correlation coefficient was low to moderate. Therefore, the SSEP features are all correlated with each other, but the correlation between the amplitude and TFA power was higher than that between the latency (Table 2).

**Table 2:** Correlation coefficient assessment between com*p*ression and SSEP features and between features of each other.

All subjects (N = 136)	Left amplitude (τ) *p*-value	Right amplitude (τ) *p*-value	Left latency (τ) *p*-value	Right latency (τ) *p*-value	Left TFA power (τ) *p*-value	Right TFA power (τ) *p*-value
CCR	0.124* 0.033	0.118* 0.042	− 0.053 0.361	− 0.082 0.159	0.176** 0.0002	0.170** 0.003
MSCC	− 0.161** 0.006	− 0.172** 0.003	0.109 0.061	0.133* 0.022	− 0.150** 0.010	− 0.175** 0.003
Left Amplitude	1.000 −	0.445** 0.000	− 0.408** 0.000	− 0.223** 0.000	0.698** 0.000	0.419** 0.000
Right Amplitude		1.000 −	− 0.220** 0.000	− 0.330** 0.000	0.433** 0.000	0.699** 0.000
Left Latency			1.000 −	0.534** 0.000	− 0.352** 0.000	− 0.225** 0.000
Right Latency				1.000 −	− 0.224** 0.000	− 0.283** 0.000
Left TFA power					1.000 −	0.555** 0.000
Right TFA power						1.000 −

(τ): Kendall’s tau-b correlation coefficient; significant differences are expressed as *p*-values, *p-value < 0.05; **p -value < 0.01.

### Different amplitude abnormalities and power loss were estimated at each MRI grade

**Figure 4:**
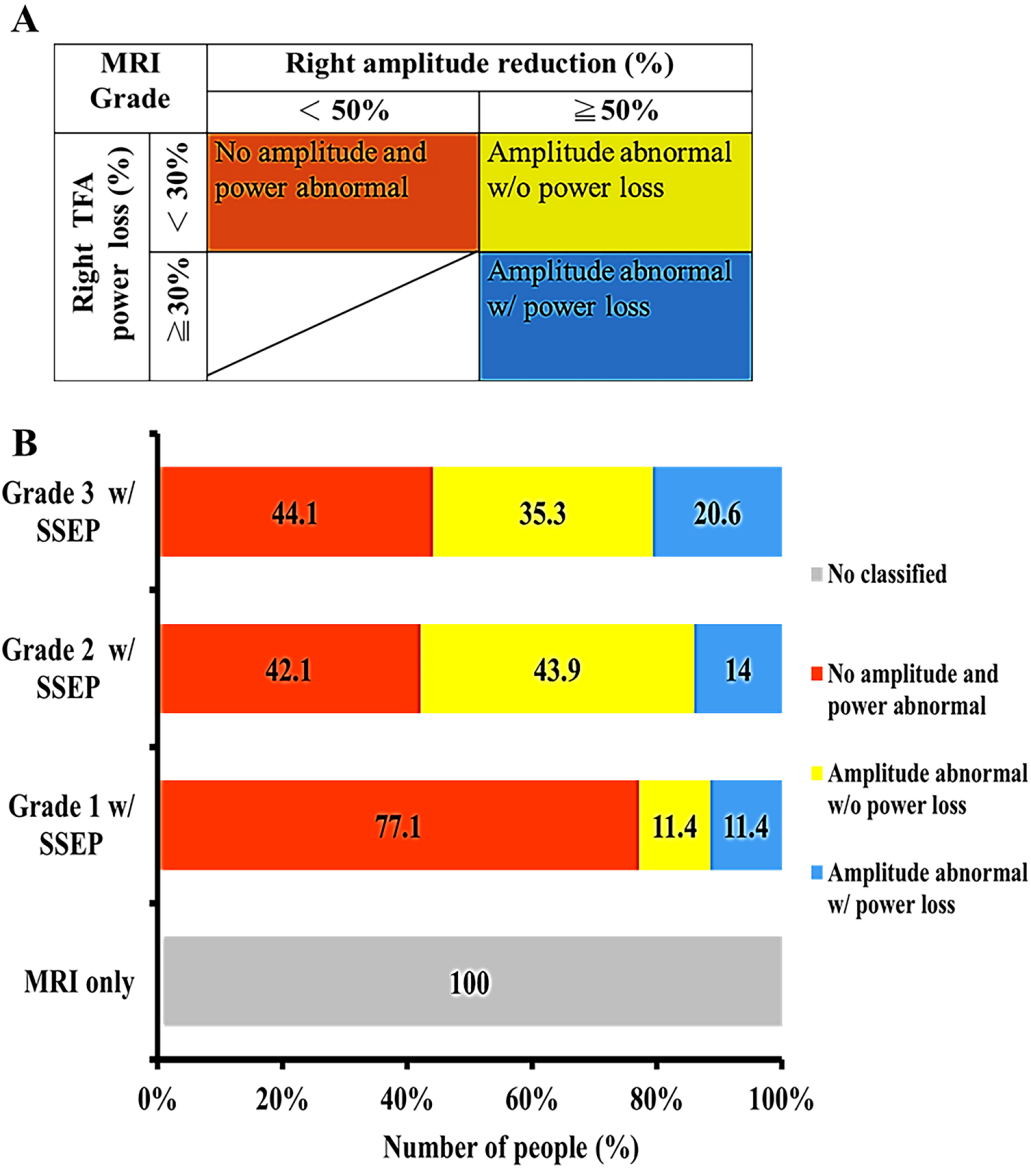
Quantification of integrated results to speculate SCC progression. The results of changes in SSEP features under the same and different MRI grades are shown in Fig. 4. Within each MRI grade, three types of neurological function differences were inferred from changes in amplitude and TFA power (**A**). Previously ungraded MRI (Fig. 4B, MRI only) could not provide relevant information on neurological function, and the integration of MRI with SSEP features in this study may provide data on possible SCC progression (**B**). When MRI grades were higher, the proportion of patients without amplitude and TFA power abnormalities decreased, the number of patients with amplitude abnormalities with or without power loss increased, and changes in TFA power could be used as an indicator of further neurological deterioration.

Based on the statistical difference and feature importance between the MRI grade and right-side amplitude and TFA power (Table 1, Fig. 3), the respective mean values of the right-side amplitude (2.58 ± 0.40 standard error of the mean) and TFA power (6.37 ± 1.43 standard error of the mean) in the control group were compared to calculate the percentage change in amplitude reduction and power loss in the SCC group. As a result, three changes in amplitude abnormality and power loss in each MRI grade were estimated based on the criteria of 50% (inclusive) reduction in amplitude and 30% (inclusive) in power loss and were significantly different between MRI grade groups (*p*-value < 0.05). However, if the amplitude is reduced by < 50%, no TFA power loss occurs (Table 3, Fig. 4A). First, the amplitude change was < 50% and the TFA power change was < 30%, indicating that there were no amplitude and power abnormality. Moreover, 77.1% of the patients with SCC had MRI grade 1, 42.1% had MRI grade 2, and 44.1% had MRI grade 3. Second, the amplitude change was ≧50%, and the TFA power change was < 30%, indicating amplitude abnormality, but there was no power loss. At this stage, 11.4% of the patients had MRI grade 1, 43.9% had MRI grade 2, and 35.3% had MRI grade 3. Third, the amplitude change was ≧50% and the TFA power change is ≧30%, indicating amplitude abnormality and accompanying power loss. At this stage, 11.4% of the patients had MRI grade 1, 14% had MRI grade 2, and 20.6% had MRI grade 3. Therefore, the estimated three changes in amplitude and power abnormalities may help in identifying differences in neurological impairment in patients with SCC at the same MRI grade.

**Table 3:** Differences in the degree of neurological impairment in patients with SCC are differentiated by changes in SSEP features under each MRI grade.

Grade 1 (n = 35)	Right amplitude reduction (%)		*p*-value
	< 50% (n = 27)	≧ 50% (n = 8)	
Right TFA power loss < 30% (n = 31)	27 (27/35 = 77.1%)	4 (4/35 = 11.4%)	0.001**
Right TFA power loss ≧30% (n = 4)	0 (0/35 = 0%)	4 (4/35 = 11.4%)	

Grade 2 (n = 57)	Right amplitude reduction (%)		*p*-value
	< 50% (n = 24)	≧ 50% (n = 33)	
Right TFA power loss < 30% (n = 49)	24 (24/57 = 42.1%)	25 (25/57 = 43.9%)	0.016*
Right TFA power loss ≧30% (n = 8)	0 (0/57 = 0%)	8 (8/57 = 14%)	

Grade 3 (n = 34)	Right amplitude reduction (%)		*p*-value
	< 50% (n = 15)	≧ 50% (n = 19)	
Right TFA power loss < 30% (n = 27)	15 (15/34 = 44.1%)	12 (12/34 = 35.3%)	0.011*
Right TFA power loss ≧30% (n = 7)	0 (0/34 = 0%)	7 (7/34 = 20.6%)	

Significant differences are expressed as *p*-values; *p-value < 0.05; **p-value < 0.01.

### Integrating MRI grading and SSEP features to infer possible SCC progression

Clinically, the MRI findings did not correlate with the neurological function of the patients (Fig. 4B, MRI only). Therefore, this study graded MRI scans and quantified patient distribution under the same and different MRI grades (Fig. 4B). The integrated results showed that in MRI grade 1, 77.1% of the patients did not have amplitude and power abnormality, and when the MRI grade was higher, the proportion of patients decreased to 42.1%–44.1%, a decrease of about 1.8 times. At MRI grade 1, the proportion of patients with amplitude abnormalities without power loss was 11.4%, increasing to 35.3%–43.9% at higher MRI grades. The proportion of patients with amplitude abnormalities with power loss also increased from 11.4% to 14%–20.6%. The overall proportion (55.9%–57.9%) of patients with MRI grade 2–3 amplitude abnormalities (with or without power loss) was approximately 2.4 times higher than those with MRI grade 1 (22.8%). In addition, among patients with amplitude abnormalities, a difference was found between those with and without power loss, indicating that the power change should be after the abnormal amplitude display. Therefore, the power change can be used as an indicator of the re-deterioration of neurological function. From the results quantifying the patient distribution, even if the SCC level in the patients is the same, a difference in neurological functions exists in patients. With increased SCC levels, the significantly visible distribution ratio indicated that the disease gradually tended to cause neurological deterioration because SCC is irreversible unless there is surgical intervention. Therefore, the system integrating MRI grade and SSEP features tracks the changes in neurological function in patients with SCC under the same and different compression levels through the changes in amplitude and power.

## Discussion

For SCC diagnosis, timely identification of clinical features and understanding of its progression to inform clinical decision-making is critical. In addition to the use of SSEP for an objective measurement of neurological function in patients with SCC, Xiaoning et al. predicted the association of progressive pathological development with neurological deterioration in mild cervical spondylotic myelopathy by categorical SSEP^35^. However, this study focused on the integration of MRI scans and SSEP features in patients with SCC. Our results show that the higher the SCC level, the higher the MRI grade. Under the same and different compression grades, the amplitude and TFA power of SSEP features can be used to infer different degrees of abnormality, and the risk for SCC progression can be inferred according to the distribution results of the patient. When MRI Grade 1 develops to Grade 2, it can be seen that the population distribution of no amplitude and power abnormal is significantly reduced, while amplitude abnormal without power loss is significantly increased, which shows that the relative neurological function is further changed by compression. Since neurological abnormalities are irreversible, when the compression progresses from Grade 2 to Grade 3, the distribution of amplitude abnormal without power loss decreases, while amplitude abnormal with power loss increases from 14 to 20.6%, indicating that when the compression progresses from Grade 2 to Grade 3, neurological function is already abnormal or even deterioration. Therefore, the integration of MRI grading and SSEP data can provide clinicians with a patient's current disease progression and provide more appropriate medical decisions. However, the findings of this study agree with those of Xiaoning et al. that SSEP abnormalities indicate neurological decline and may reflect myelopathy worsening.

Kang et al. evaluated an MRI grading system to provide a reliable assessment of the spinal stenosis level and showed the usefulness of such a grading system in clinical settings. Although Kang et al. found a crude positive association between higher MRI grades and the number of people with worsening neurological function, owing to the study limitations, the extent of neurological function deterioration was not investigated further^40^. Therefore, based on the findings of Kang et al., this study further provides a way to integrate the MRI grades with preoperative SSEP features to meet the needs of clinical diagnosis. Our findings suggest that MRI grading is a reliable assessment of cervical and thoracic stenosis. Integrating the advantages of MRI grading and SSEP features provides an opportunity to understand the risk for disease progression and possible to quantify the distribution of patients with SCC having different degrees of neurological changes under the same and different compression levels, which may be used to reasonably explain why previous studies found that even with evidence of compression on MRI, most patients (approximately 75%) remained stable; however, some showed progressive disease or a rapidly worsening condition^11^. Most previous studies on MRI have used quantitative measures of CCR and MSCC. However, our study elucidates that the correlation between CCR and MSCC, and SSEP, is low. Therefore, physical structural information derived from these quantitative measurements may not be sufficiently correlated with changes in neurological function.

In surgeries such as spinal injuries/deformities, motor evoked potentials (MEPs) are useful additions to SSEP for real-time monitoring, with detection sensitivity and specificity of up to 100%^41,42^ and are used to assess motor function. In comparison to SSEP, MEP has certain limitations that make it more restricted in use. For instance, MEP requires constant current or voltage stimulation of the brain motor cortex, which often causes strong contractions of the scalp and facial muscles, resulting in discomfort^43^. Secondly, MEP records are periodic rather than continuous, which limits its ability to detect changes in real-time. Thirdly, while transcranial magnetic stimulation (TMS) can replace the current method of MEP stimulation, there is a risk of inducing seizures, making it unsuitable for patients with metal implants in their brain or heart^44^*.*

As this study found that SCC-associated SSEP featured a reduction in amplitude and power loss, but no latency delay was found, it was presumed to be related to the exclusion of diabetes, renal insufficiency, and acute and chronic neuritis that induce peripheral neuropathy. Previous studies have shown that peripheral neuropathy can lead to abnormal myelin sheaths, resulting in decreased nerve conduction velocity, and its neurophysiological manifestation is prolonged latency^19,28,45,46^. Therefore, further studies are needed to investigate the differences in latency delay between patients with SCC alone and those with peripheral neuropathy with SCC. However, this study has some limitations. First, this is a retrospective study, and missing medical record data may limit our assessment. For example, due to the retrospective design of this study and the diversity of MRI image sources collected, MRI imaging acquisition parameters and equipment may differ, and we cannot provide detailed parameters and technical information for all MRI images. It is worth noting that the MRI grading in this study was performed by a neurologist and based on a reliable assessment system for cervical spinal stenosis provided by Kang et al.'s research. Second, this study was conducted in one center and included a small number of samples, which may limit its generalizability; thus, further multicenter studies are needed. Third, SSEP can be used to assess the presence of neuropathy caused by compression in the entire conduction path of the peripheral nervous system to the central nervous system, so it may not be suitable for single-segment spine examination. Fourth, although the present study did not directly investigate dMRI/DTI, previous research has demonstrated the potential benefits of these techniques in predicting disease progression^20^ or quantifying the extent of spinal cord injury^21^, whether through quantifying local spinal cord microstructural structures or findings of MRI- Eps^19^. However, due to our institution's lack of inclusion of dMRI/DTI in routine examinations of SCC patients, but rather primarily used in the examination of ischemic stroke and brain lesion patients, we currently lack sufficient data to verify the potential benefits of these techniques for this study.

## Conclusions

For SCC, MRI can provide physical structural information, whereas SSEP can provide neural functional information; however, there is no integrated method that can combine the advantages of both. In this study, MRI scans were graded, and the correlation between each MRI grade and change in SSEP features was investigated. This study found that integrating the amplitude and TFA power changes of SSEP features with MRI grading results may help speculate progression at the same and different SCC levels, and hopefully contribute to the diagnosis of SCC.

## Methods

### Inclusion and exclusion criteria

This retrospective study collected the SSEP data of 136 patients undergoing cervical or thoracic spine surgery with intraoperative neurological monitoring (IONM; 16 channels; Cadwell, Cascade Elite) equipment in the operating room of National Taiwan University Hospital from 2007 to 2020; there was no sex-based recipient selection. Patients aged 20–69 years who have preoperative (before any surgical incision) and during surgery (after any surgical incisions) SSEP records and axial and sagittal T2-weighted MRI records and no previous history of surgical treatment for myelopathy were included. Patients who have epilepsy, brain disease, brain injury, neurological and psychiatric diseases, or have previous history of surgical treatment for myelopathy; medical records indicate taking antidepressants or sleeping pills; patients with non-cervical or thoracic SCC lesions; have peripheral neuropathy (caused by diabetes, renal failure, neuropathy, nerve infection, and other diseases); have no complete SSEP records; and have acute traumatic spinal cord damage were excluded. In total, 85 male and 51 female patients were recruited, with an average age of 54 (range, 21–69) years; average height, 175 (range, 146–183) cm; and average weight, 68 kg (range 44–101 kg; the body weight of one patient not available in the medical records). Ten patients in the control group (6 men, 4 women) were clinically diagnosed with spinal diseases but had no MRI-confirmed SCC. The SCC group included 126 patients (79 men, 47 women) with only one lesion clinically diagnosed and confirmed by MRI.

This study protocol was approved by the Institutional Review Board of National Taiwan University Hospital (NTUH) Hsin-Chu Branch with ID 110–059-E, which also waived the need for informed consent, as the study was retrospective and used de-identified data. All methods were performed in accordance with the relevant guidelines and regulations.

### MRI grading

Referring to previous studies of MRI grading systems^40,47^, to provide a reliable assessment of spinal stenosis, neurosurgeons assisted in classifying sagittal T2-weighted MRI scans into grades 0–3 according to the changes in the subarachnoid space and signal on MRI scans. Grade 0 was defined as subarachnoid occlusion < 50%, with normal cerebrospinal fluid (CSF) space around the spinal cord and no evidence of spinal cord malformation or intraspinal signal changes (control group). Grade 1 indicated that > 50% CSF occlusion in the subarachnoid space, and there was no obvious spinal cord deformity. Grade 2 indicated that the spinal cord was compressed and deformed significantly, but the MRI scan signal does not change. Grade 3 indicated marked spinal cord narrowing and changes in the MRI scan signal. Clinically, quantitative measures are used to assess the physically compressed structure of the spinal cord^12,48^, including the spinal cord compression ratio (CCR) and maximum spinal cord compression (MSCC). A lower CCR or higher MSCC ratio indicates a higher level of SCC and deformation. In this study, Image J software was used to evaluate axial and sagittal T2-weighted MRI scans and calculate the CCR and MSCC ratio to determine whether MRI grading could reveal differences in the level of physical SCC.

### SSEP data collection

In clinical surgery, IONM equipment has been used to continuously record changes in electrophysiological signals^41,49,50^. In our study, SSEP monitoring data was obtained from the IONM equipment at the National Taiwan University Hospital. The IONM system was used to repeatedly stimulate the median nerve of the upper extremity and the posterior tibial nerve of the lower extremity to elicit SSEPs, which recorded the corresponding nerve signals N20 and P40 on the scalp and detected relevant physiological responses. Since our experimental design aimed to examine SSEP features under compression at both the Cervical and Thoracic regions of the spinal cord, we collected SSEP data from stimulation of the posterior tibial nerve of the lower extremity. In IONM equipment settings, the electrical stimulation time interval between the left and right sides is approximately 2 min; stimulation intensity, 22 mA; pulse width, 0.2 ms; and stimulation frequency, 2.66 Hz/s. With sampling at 200 Hz and passing through 10–500 Hz bandpass filters, the tibial nerve analysis time was 100 ms, and the electrode impedance was kept below 5 kiloohms (kΩ). Electrodes to detect SSEP were positioned according to the International 10–20 system and placed at the vertex Cz' of the scalp (anode, 2 cm posterior to the Cz of the 10–20 system) and midfrontal at Fpz (cathode; reference electrode). Serial SSEP electroencephalography (EEG) recordings were collected by repeated electrical stimulations of the posterior tibial nerves of the left and right ankle joints, and SSEP recordings before any surgical incision were captured as preoperative data.

### SSEP feature extraction

In recent years, given the massive and structured clinical data, the Matlab Signal Processing Toolbox has been used for preprocessing and feature extraction of MRI scans and electrophysiological potential data ^19,51,52^. In this study, MATLAB Signal Processing Toolbox (version R2021a; MathWorks, Natick, MA, USA) was used to extract amplitude and latency delay features from the raw SSEP signal data for each subject. The time vector was measured in milliseconds (taken from a sampling time of 100 ms for tibial data) and the amplitude was measured in microvolts on the Y-axis. The left and right tibial data were averaged separately to produce an image, and the highest and lowest amplitudes were found on the image. The difference between the highest and lowest amplitudes on the Y-axis was used to measure the amplitude change, and the X-axis value of the highest amplitude was used to measure the latency period. The ERP feature was calculated as:

$$\mathrm{ERP}=\frac{\sum_{n=1}^{N}{X}_{n}}{N}$$

where *N* is the number of trail and *X* is the time-series EEG signal.

After subtracting the average amplitude and delay measurements of the control group on the left and right sides, the criteria for judging neurological impairments were changes in the amplitude potential decreased by 50% (inclusive) and/or latency delay increased by > 10% (inclusive) of the preoperative SSEP ERP response wave^49,53–55^. Additionally, MATLAB was used to decompose the preoperative SSEP into three parameters: time, spectrum, and power. TFA was performed using MATLAB spectrogram function, where the time-series EEG data were subjected to short-time Fourier transform (STFT)^56^. The TFA feature was computed as:

$$\mathrm{STFT}\left\{x\left(t\right)\right\}\left(\tau ,\omega \right)={\int }_{-\infty }^{\infty }x\left(t\right)w(t-\tau ){e}^{-i\omega t}dt$$

where *x(t)* is the time-series EEG data, *ω* is the frequency, and *w(τ)* is the short-time window function.

The generated time–frequency image was presented in a two-dimensional graph, and the power intensity was represented by color. Previous studies have shown that spinal cord injury may result in a wider distribution of power information, so in this study, a fixed area range was programmed using MATLAB, with the X-axis representing time (100 ms) and the Y-axis representing frequency (measured in Hz), to calculate the power lost by each SCC patient within this fixed area range. The average TFA value in the fixed area range for the control group was used as a reference, and a > 30% decrease in power value for the experimental group was considered as an abnormal power loss standard^37,38^. Finally, the MATLAB tool displays the results of integrating MRI grading and SSEP features and generates .mat and .xls files containing the extracted features, which can be imported into MATLAB for analysis or Microsoft Excel for further offline processing.

### Statistical analysis

All statistical programs were performed using IBM SPSS Statistics for Windows, version 20.0 (IBM Corp., Armonk, NY, USA). Crosstabs in descriptive statistics were used to explore differences between MRI grades and sex categorical variables, Fisher’s exact test was used to express the probability of correctness, and a significant linear correlation was indicated as *P* < 0.05 or *P* < 0.01. Given the small number of cases in the sample group (control group, < 30 cases), it followed a non-normal distribution, so the nonparametric statistical test was used. The median (Q2; second quartile) of the data was used as the representative value of the research data to explore the differences between MRI grades and age, height, weight, CCR, MSCC, and SSEP feature variables. The Mann–Whitney U test was used to analyze two independent samples, the Kruskal–Wallis H coefficient was used for three or more independent samples, and *P* < 0.05 and *P* < 0.01 were considered significant. The Kendall's tau_b (τ) test correlation coefficient, the significance using two-tailed test, was used to detect the correlation between the CCR and MSCC and three SSEP features and the correlation of the three SSEP features with each other; *P* < 0.05 and *P* < 0.01 were considered significant. The correlation coefficient (τ) was between − 1 and + 1; τ < 0.3 was a low correlation; 0.3–0.7, moderate correlation; > 0.7, high correlation. Given the inability to analyze the relationship between multiple categorical variables and other continuous variables with SPSS, SSEP feature importance analysis was performed using QOCA aim^57^ (Quanta Omni Cloud Care for AI Medical Platform, QOCA Aim version 2.0). The classification model was built by the implementation of machine learning CART LogisticRegression^58^. Machine learning training was conducted with 80% of the sample size, and each validation and test were carried out with a ratio of 10%. The higher the weighting of the results obtained by MRI grading and SSEP features, the higher the importance of the feature.

## Supplementary Information

Supplementary Information 1.

Supplementary Information 2.

Supplementary Information 3.

## Data Availability

All data supporting the findings of this study are available in the article and its supplementary materials.

## Code availability

All in-house implemented computer codes are available in supplementary document.
