# Supplementary material for: Integration of MRI and somatosensory evoked potentials facilitate diagnosis of spinal cord compression
Source: Sci Rep. 2023 May 15;13:7861. doi: 10.1038/s41598-023-34832-2 (PMC10185544; doi:10.1038/s41598-023-34832-2)
Supplement: Supplementary file 1 — Supplementary Information 1. [file 41598_2023_34832_MOESM1_ESM.docx]

**Title:**

Integration of MRI and somatosensory evoked potentials facilitate diagnosis of spinal cord compression

**Authors:**

Shu-Pin Sun, ^1,2,5^ , Chun-Ren Phang, ^1,5^ , Shey-Cherng Tzou, ^3,7^ , Chang-Mu Chen, MD, Ph.D.^4^,^*^ , Li-Wei Ko, Ph.D.^1,5,6,7^,^*^

**Affiliations:**

^1^ International Ph.D. Program in Interdisciplinary Neuroscience (UST), College of Biological Science and Technology, National Yang Ming Chiao Tung University, Hsinchu 300, Taiwan.

^2^ Department of Medical Research, National Taiwan University Hospital Hsin-Chu Branch, Hsinchu 300, Taiwan.

^3^ Institute of Molecular Medicine and Bioengineering, National Yang Ming Chiao Tung University, Hsinchu 300, Taiwan.

^4^ Department of Surgery, College of Medicine and Hospital, National Taiwan University, Taipei 100, Taiwan.

^5^ Center for Intelligent Drug Systems and Smart Bio-devices (IDS^2^B), College of Biological Science and Technology, National Yang Ming Chiao Tung University, Hsinchu 300, Taiwan.

^6^ Institute of Electrical and Control Engineering, Department of Electronics and Electrical Engineering, National Yang Ming Chiao Tung University, Hsinchu 300, Taiwan.

^7^ Department of Biomedical Science and Environment Biology, and the Drug Development and Value Creation Research Center, Kaohsiung Medical University, Kaohsiung 807, Taiwan.

*** Corresponding author:**

Dr. Chang-Mu Chen and Dr. Li-Wei Ko both are the corresponding author. Dr. Chang-Mu Chen current address: No. 7, Zhongshan South Road, Taipei 10002, Taiwan, ROC, Email: [cmchen10@ms27.hinet.net](mailto:cmchen10@ms27.hinet.net). Dr. Li-Wei Ko current address: 734, Engineering Bldg. 5, 1001 Daxue Road, Hsinchu 30010, Taiwan, ROC, Email: [lwko@nycu.edu.tw](mailto:lwko@nycu.edu.tw).

**MATLAB R2021a cord**

| **Computer code for SSEP features analysis: amplitude and latency** |
| --- |
| patient_number='';  sub=;    % Load data  load('C:\Users\pin\Desktop\EEG data\patient data\20221120\allsub.mat');  tempdir='C:\Users\pin\Desktop\EEG data\patient data\20221120\';  direc=strcat(tempdir,patient_number,'\');  filename{1}=dir(strcat(direc,patient_number,'_Lt Tibial*.csv'));  filename{2}=dir(strcat(direc,patient_number,'_Rt Tibial*.csv'));    % Align  for i=1:2  raw{sub,i}=csvread(strcat(filename{i}.folder,'\',filename{i}.name),10,1);  n(i)=size(raw{sub,i},2);  raw_line{sub,i}=reshape(raw{sub,i},640*n(i),1);  end    % ERP  figure,  for i=1:2  erp{sub,i}=mean(raw{sub,i},2);  ax(i)=subplot(1,2,i); plot([1:640]/6400*1000,erp{sub,i});  [delaytime{sub,i}(1),delaytime{sub,i}(2)]=max(erp{sub,i}(65:end));  delaytime{sub,i}(2)=(delaytime{sub,i}(2)+64)/6400;  xlabel('time (ms)');  end    raw{sub,3}=patient_number;  erp{sub,3}=patient_number;  delaytime{sub,3}=patient_number;    % Save  save('C:\Users\pin\Desktop\EEG data\patient data\20221120\allsub.mat','delaytime','raw','erp');    %%    for i=1:2  figure;  for j=1:size(raw{sub,i},2)  subplot(5,8,j), plot(raw{sub,i}(:,j));  end  end |

| **Computer code for SSEP features analysis: TFA power** |
| --- |
| patient_number='';  sub=;    % Load data  %load('C:\Users\pin\Desktop\EEG data\patient data\20221120\allsub_TFA.mat');  tempdir='C:\Users\pin\Desktop\EEG data\patient data\20221120\';  direc=strcat(tempdir,patient_number,'\');  filename{1}=dir(strcat(direc,patient_number,'_Lt Tibial*.csv'));  filename{2}=dir(strcat(direc,patient_number,'_Rt Tibial*.csv'));    % Align  for i=1:2  raw{sub,i}=csvread(strcat(filename{i}.folder,'\',filename{i}.name),10,1);  n(i)=size(raw{sub,i},2);  raw_line{sub,i}=reshape(raw{sub,i},640*n(i),1);  end    % Spectrogram  for i=1:2  s2{sub,i}=[];  n(i)=size(raw{sub,i},2);  for m=1:n(i)  [s{sub,i},f,t] = spectrogram(raw{sub,i}(:,m),64,[],[],6400);  s2{sub,i}=cat(3,s2{sub,i},real(s{sub,i}));  end  TFA{sub,i}=mean(abs(s2{sub,i}),3);  figure, imagesc(TFA{sub,i}(1:21,2:end),[0 40]); colorbar;  xticks(1:18); xticklabels(t(2:end)*1000);  yticks(1:21); yticklabels(f(1:21));  ylabel('frequency (hz)'); xlabel('time (ms)');  end    TFA{sub,3}=patient_number;  save('C:\Users\pin\Desktop\EEG data\patient data\20221120\allsub_TFA.mat','TFA','t','f'); |

| **Computer code for SSEP features analysis: TFA power average** |
| --- |
| %% Healthy  load('C:\Users\pin\Desktop\EEG data\patient data\20221120\allsub_TFA.mat');  temp=zeros(129,19);  for sub=1:10  for i=1:2  temp=TFA{sub,i}(1:129,1:19)+temp;  end  end  healthy_TFA=temp/22;  figure, imagesc(healthy_TFA(1:21,2:18),[0 40]); colorbar;  xticks(1:17); xticklabels(t(2:18)*1000);  yticks(1:21); yticklabels(f(1:21));  ylabel('frequency (hz)'); xlabel('time (ms)');    % Fill in  x1 = 6; y1 = 1;  x2 = 13; y2 = 6;    temp_TFA=healthy_TFA(y1:y2,x1+1:x2+1);  mean_TFA=mean(mean(temp_TFA));    %% Compression    load('C:\Users\pin\Desktop\EEG data\patient data\20221120\allsub_TFA.mat');  temp=zeros(129,19);  for sub=20:81  for i=1:2  patient_TFA(sub-19,i)=mean(mean(TFA{sub,i}(y1:y2,x1+1:x2+1)));  end  end  TFA_loss=((patient_TFA*100)/mean_TFA)-100;  save('TFA_loss.mat','TFA_loss'); |
