# Supplementary material for: Integration of MRI and somatosensory evoked potentials facilitate diagnosis of spinal cord compression
Source: Sci Rep. 2023 May 15;13:7861. doi: 10.1038/s41598-023-34832-2 (PMC10185544; doi:10.1038/s41598-023-34832-2)
Supplement: Supplementary file 2 — Supplementary Information 2. [file 41598_2023_34832_MOESM2_ESM.docx]

**Title:**

Integration of MRI and somatosensory evoked potentials facilitate diagnosis of spinal cord compression

**Authors:**

Shu-Pin Sun, ^1,2,5^ , Chun-Ren Phang, ^1,5^ , Shey-Cherng Tzou, ^3,7^ , Chang-Mu Chen, MD, Ph.D.^4^,^*^ , Li-Wei Ko, Ph.D.^1,5,6,7^,^*^

**Affiliations:**

^1^ International Ph.D. Program in Interdisciplinary Neuroscience (UST), College of Biological Science and Technology, National Yang Ming Chiao Tung University, Hsinchu 300, Taiwan.

^2^ Department of Medical Research, National Taiwan University Hospital Hsin-Chu Branch, Hsinchu 300, Taiwan.

^3^ Institute of Molecular Medicine and Bioengineering, National Yang Ming Chiao Tung University, Hsinchu 300, Taiwan.

^4^ Department of Surgery, College of Medicine and Hospital, National Taiwan University, Taipei 100, Taiwan.

^5^ Center for Intelligent Drug Systems and Smart Bio-devices (IDS^2^B), College of Biological Science and Technology, National Yang Ming Chiao Tung University, Hsinchu 300, Taiwan.

^6^ Institute of Electrical and Control Engineering, Department of Electronics and Electrical Engineering, National Yang Ming Chiao Tung University, Hsinchu 300, Taiwan.

^7^ Department of Biomedical Science and Environment Biology, and the Drug Development and Value Creation Research Center, Kaohsiung Medical University, Kaohsiung 807, Taiwan.

*** Corresponding author:**

Dr. Chang-Mu Chen and Dr. Li-Wei Ko both are the corresponding author. Dr. Chang-Mu Chen current address: No. 7, Zhongshan South Road, Taipei 10002, Taiwan, ROC, Email: [cmchen10@ms27.hinet.net](mailto:cmchen10@ms27.hinet.net). Dr. Li-Wei Ko current address: 734, Engineering Bldg. 5, 1001 Daxue Road, Hsinchu 30010, Taiwan, ROC, Email: [lwko@nycu.edu.tw](mailto:lwko@nycu.edu.tw).

**Spinal cord compression patient’s main and secondary pathological diagnosis:**

| **Main pathological diagnosis** | **Patients Number** | **Secondary pathological diagnosis** |
| --- | --- | --- |
| spondylosis | 120 | Herniated intervertebral disc (HIVD) |
|  |  | Ossification of posterior longitudinal ligament (OPLL) |
|  |  | spinal stenosis |
|  |  | spine disorder with radiculopathy |
|  |  | spine fracture |
| Spinal tumor | 16 | metastasis |
|  |  | intradural extramedullary tumor |
|  |  | intraspinal intramedullary tumor |

**Spinal cord compression patient’s main and secondary pathological diagnosis detail:**

| **Patients number** | **Main pathological diagnosis** | **Secondary pathological diagnosis** |
| --- | --- | --- |
| 1 | Spondylosis | Herniated intervertebral disc (HIVD) with cord compression |
| 2 | Spondylosis | HIVD with cervical spondylotic myelopathy |
| 3 | Spondylosis |  |
| 4 | Spinal tumor |  |
| 5 | Spondylosis | Cervical disc disorder with radiculopathy |
| 6 | Spondylosis |  |
| 7 | Spondylosis |  |
| 8 | Spondylosis | HIVD |
| 9 | Spondylosis | HIVD |
| 10 | Cervical spine fracture |  |
| 11 | Spondylosis | HIVD |
| 12 | Spondylosis | Spondylosis and spinal stenosis with OPLL and retrolisthesis |
| 13 | Spondylosis | Ossification of posterior longitudinal ligament (OPLL) |
| 14 | Spinal tumor |  |
| 15 | Spondylosis | HIVD with stenosis |
| 16 | Spinal tumor |  |
| 17 | Spondylosis |  |
| 18 | Spondylosis | Dislocation of C4/C5 cervical vertebrae, subsequent encounter(Herniated intervertebral disc ) |
| 19 | Spondylosis | HIVD |
| 20 | Spinal tumor | intradural extramedullary tumor |
| 21 | Bone metastasis | Pancreatic cancer with C6 metastasis |
| 22 | Spondylosis | OPLL |
| 23 | Spine bone metastasis |  |
| 24 | OPLL |  |
| 25 | Spinal stenosis |  |
| 26 | Spondylosis | HIVD |
| 27 | Spondylosis | HIVD |
| 28 | Spinal stenosis |  |
| 29 | Spinal stenosis | HIVD with cord compression |
| 30 | Spondylosis | HIVD |
| 31 | Spondylosis | HIVD |
| 32 | OPLL |  |
| 33 | Spondylosis | HIVD |
| 34 | Spondylosis |  |
| 35 | Spondylosis |  |
| 36 | Spinal stenosis |  |
| 37 | Spondylosis | HIVD |
| 38 | OPLL |  |
| 39 | Spinal stenosis | HIVD |
| 40 | Spondylosis |  |
| 41 | OPLL |  |
| 42 | Myelopathy in diseases classified elsewhere |  |
| 43 | Spondylosis |  |
| 44 | Spondylosis | HIVD |
| 45 | Spondylosis | HIVD |
| 46 | Spondylosis |  |
| 47 | Spondylosis | spondylosis with OPLL, complicated with spinal cord compression |
| 48 | HIVD |  |
| 49 | Cervical myelopathy | HIVD |
| 50 | Spondylosis | HIVD |
| 51 | Dislocation of C5/C6 cervical vertebrae |  |
| 52 | OPLL |  |
| 53 | Spondylosis | Spondylosis with spinal canal stenosis |
| 54 | Spondylosis | HIVD |
| 55 | Spinal stenosis |  |
| 56 | OPLL | Spinal stenosis, cervical region |
| 57 | Spondylosis | HIVD |
| 58 | Thoracic myelopathy | T2-3 intraspinal extradural tumor |
| 59 | HIVD |  |
| 60 | Spondylosis myelopathy | with spinal cord compression |
| 61 | Cervical myelopathy | HIVD |
| 62 | Spondylosis with myelopathy | HIVD |
| 63 | Spondylosis |  |
| 64 | Spondylosis |  |
| 65 | Spondylosis |  |
| 66 | Spondylosis | HIVD |
| 67 | Spondylosis | HIVD |
| 68 | Spinal stenosis | HIVD |
| 69 | Spondylosis | HIVD |
| 70 | Spondylosis |  |
| 71 | Spondylosis | HIVD |
| 72 | Spinal stenosis | HIVD |
| 73 | Spinal tumor | intradural and extramedullary |
| 74 | Spinal tumor |  |
| 75 | HIVD |  |
| 76 | Spondylosis | HIVD |
| 77 | Spinal tumor | Intraspinal extramedullary tumor, suspected left C8 neuroma |
| 78 | Cervical myelopathy | HIVD |
| 79 | Spondylosis |  |
| 80 | Spondylolisthesis, acquired | HIVD |
| 81 | Spondylosis | HIVD |
| 82 | Spondylosis | HIVD |
| 83 | HIVD |  |
| 84 | Spondylosis | OPLL with cord compression |
| 85 | Cervical disc disorder with myelopathy | HIVD |
| 86 | Cervical disc disorder with myelopathy | HIVD |
| 87 | Spondylosis | HIVD |
| 88 | Spine tumor |  |
| 89 | Spondylosis |  |
| 90 | Spondylosis | HIVD |
| 91 | Spinal tumor |  |
| 92 | Spondylosis | HIVD |
| 93 | HIVD |  |
| 94 | Spondylosis |  |
| 95 | Spondylosis |  |
| 96 | HIVD |  |
| 97 | Spondylosis | HIVD |
| 98 | Spondylosis | HIVD |
| 99 | Spondylosis fusion |  |
| 100 | Spondylosis | HIVD |
| 101 | Spinal stenosis |  |
| 102 | Spondylosis |  |
| 103 | Spinal tumor | cervical epidural neuroma |
| 104 | Spondylosis | HIVD |
| 105 | Cavernous angioma | intraspinal intramedullary cavernoma |
| 106 | Spondylosis | HIVD |
| 107 | Spondylosis | Central cord syndrome (C6) without fracture |
| 108 | Spondylosis |  |
| 109 | Spondylosis |  |
| 110 | Spondylosis |  |
| 111 | Spondylosis | HIVD |
| 112 | Spinal stenosis |  |
| 113 | Cervical myelopathy | OPLL with spinal stenosis |
| 114 | Spondylosis | HIVD |
| 115 | Spondylosis |  |
| 116 | Spinal stenosis | HIVD |
| 117 | HIVD |  |
| 118 | Spondylosis with myelopathy |  |
| 119 | OPLL |  |
| 120 | Spondylosis | HIVD |
| 121 | Spinal metastasis |  |
| 122 | HIVD | HIVD |
| 123 | Spinal tumor |  |
| 124 | OPLL |  |
| 125 | Spinal tumor |  |
| 126 | Spondylosis | with spinal cannal stenosis |
| 127 | Cervical myelopathy |  |
| 128 | Spondylosis |  |
| 129 | Spondylosis |  |
| 130 | Spinal stenosis |  |
| 131 | Spondylosis |  |
| 132 | Spondylosis | HIVD |
| 133 | Spondylosis | HIVD and spinal stenosis |
| 134 | Spondylosis |  |
| 135 | Spondylosis |  |
| 136 | Spondylosis | HIVD |
